# Subcutaneous metastases from colon cancer: a case report

**DOI:** 10.1186/1752-1947-6-212

**Published:** 2012-07-19

**Authors:** Giuseppe Lo Russo, Fabio Accarpio, Gian Paolo Spinelli, Evelina Miele, Francesco Borrini, Linda Cerbone, Valeria Stati, Natalie Prinzi, Martina Strudel, Simone Sibio, Silverio Tomao

**Affiliations:** 1 Department of Medical-Surgical Science and Biothecnology, Oncology Unit, S.Maria Goretti Hospital, Latina - University of Rome “Sapienza” Corso della Repubblica, 04100, Latina, Italy; 2 Department of Surgery Pietro Valdoni, University of Rome “Sapienza”, Viale Regina Elena 324, 00161, Rome, Italy; 3 Department of Molecular Medicine, University of Rome “Sapienza”, Viale Regina Elena 324, 00161, Rome, Italy; 4 Department of Medical Oncology, San Camillo and Forlanini Hospitals, Circonvallazione Gianicolense 87, 00152, Rome, Italy; 5 Department of Experimental Medicine, University of Rome “Sapienza”, Viale Regina Elena 324, 00161, Rome, Italy

**Keywords:** Colorectal cancer, Dukes A stage, Skin metastasis

## Abstract

**Introduction:**

Dukes A stages of colorectal cancer are rarely reported to metastasize. Subcutaneous or skin metastases from colon cancer are rare events and usually indicate widespread disease.

**Case presentation:**

We present the case of a 72-year-old Caucasian woman with Dukes A colorectal cancer at diagnosis and, three years later, a single secondary subcutaneous involvement with no other metastatic sites. The description of this case is supported by critical analysis of its clinical, radiological and pathological features. Our report illustrates that diagnosis can be difficult and controversial when relapse occurs in early stage patients and at uncommon sites.

**Conclusion:**

The unusual and aggressive course of the reported disease stresses the importance of intensive follow-up in colorectal cancer patients with good prognostic factors.

## Introduction

Colorectal carcinoma most often metastasizes to liver and lung, while subcutaneous or skin metastases are rare [1,2], accounting for only 5% of such cases [3]. The most common malignancies reported to metastasize to the skin, both in men and women, are lung and breast cancers [4].

Usually, for almost all cancers, skin involvement is synonymous with extensive disease and frequently implies a poor prognosis [5,6]. In colorectal cancer, this event occurs mostly on the incision scars, but is also seen on the skin of the extremities, head and neck, and penis [7-9]. Some authors, such as Kaufmann *et al*., have suggested two hypotheses for this metastatic involvement: the first one implies a lymphatic and hematogenous spread of disease, while the second one speculates on an implantation during surgery [10].

Ulceration, nodules, bullae or fibrotic processes are the most common signs of cutaneous metastases.

In stage Dukes A, colorectal cancer involves only the innermost lining of the colon or rectum, with minimal invasiveness in the muscular layer.

In patients with this stage of cancer, relapse is very rare [11]. Here we describe the case of a 72-year-old woman with Dukes A colorectal cancer at diagnosis, and three years later, a single secondary involvement of the subcutaneous tissue of the left paravertebral dorsal region.

## Case presentation

A 72-year-old Caucasian woman underwent a right hemicolectomy for a Dukes A colon cancer in August 2008 (staging: pT2, pN0-0/27nodes, G2). Computed tomography (CT) of her abdomen, pelvis, and chest did not show any distant metastases, so she was not treated further. Follow-up examinations were negative until August 2009 when mammography showed a suspicious lesion of the left breast. In September 2009 a left superior and external quadrantectomy was performed. Histological examination revealed breast intraductal carcinoma (staging: pTis, p N0). For this new pathology, the patient was only treated with radiotherapy. She continued her follow-up examinations for colorectal cancer. She remained asymptomatic until May 2011, with no clinical or radiological signs of recurrence. In June 2011, she presented with a painless swelling, affecting the skin of the left paravertebral dorsal region. Serum tumor markers were not increased and colonoscopy did not show any local disease relapse. A chest X-ray excluded pulmonary metastasis and an abdominal ultrasound did not detect either liver metastases or peritoneal dissemination. Therefore, an ultrasound scan of the dorsal tumefaction was performed, showing a suspected heterogeneous, solid and vascularized lesion. She underwent a radical en-bloc resection of the dorsal swelling. Histological analysis of the excised specimen revealed a metastatic poorly differentiated adenocarcinoma, most likely originating from the large bowel, which infiltrated the skin and the subcutaneous tissue.

Macroscopically, the surgical specimen consisted of a radical en-bloc resection of subcutaneous tissue (soft-tissue) of 4x2.5x2cm which contained a nodular lesion of 3.2cm diameter. The cut surface was white to yellowish white and showed areas of necrosis and hemorrhage. The excised specimen was fixed in 10% buffered formalin and embedded in paraffin. Sections of 4μm were prepared and routinely stained with hematoxylin and eosin. Further sections were examined immunohistochemically using a streptavidin-biotin complex technique (DAKO). Prediluted antibodies against the following antigens were used: keratin 20 protein (monoclonal, 1:50; Zymed®), keratin 7 (monoclonal, 1:50; Zymed®), Thyroid transcription factor 1 (TTF-1) (monoclonal, 1:100; Zymed®), anti-estrogen receptor (monoclonal, 1:50; DAKO), and anti-progesterone receptor (monoclonal, 1:50; DAKO).

Microscopically, the tumor was characterized by nests and islands of epithelial cells with only scattered abortive glands. The cells had atypical nuclei, with evidence of nucleoli, abundant eosinophilic cytoplasm without sharply defined outlines. The inflammatory infiltrate was marked and there were hemorrhagic foci and areas of necrosis. The mitosis was either frequently or sometimes infrequently atypical (sFigure 1). The cells were focally positive for keratin 20 (Figure 2). Keratin 7, TTF1, estrogen and progesterone receptors were negative. The morphologic aspects and the immunohistochemical results were considered specific for a colonic origin. In this setting, the neoplasia was also evaluated for K-ras mutation and the molecular analysis showed the absence of mutation on codon 12 and 13 (wild type). The total body CT revealed no evidence of visceral distant metastases but showed suspicious abdominal colliquative lymphadenopathy. The patient is being treated with FOLFIRI ( leucovorin (folinic acid), fluorouracil, irinotecan hydrochloride) + bevacizumab and she remains asymptomatic.

**Figure 1:**
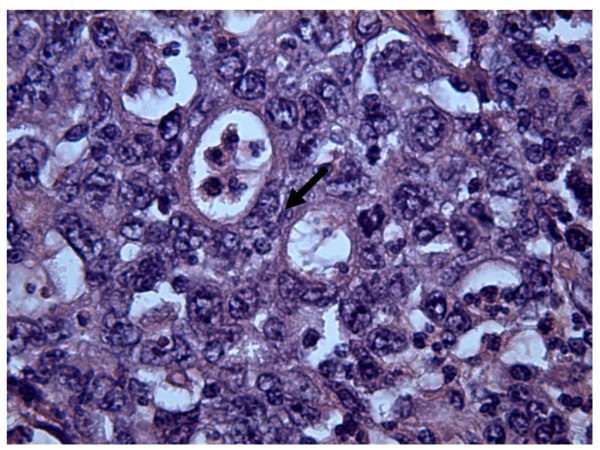
Hematoxylin/Eosin (40×): Abortive glands and atypical mitosis (arrow).

**Figure 2:**
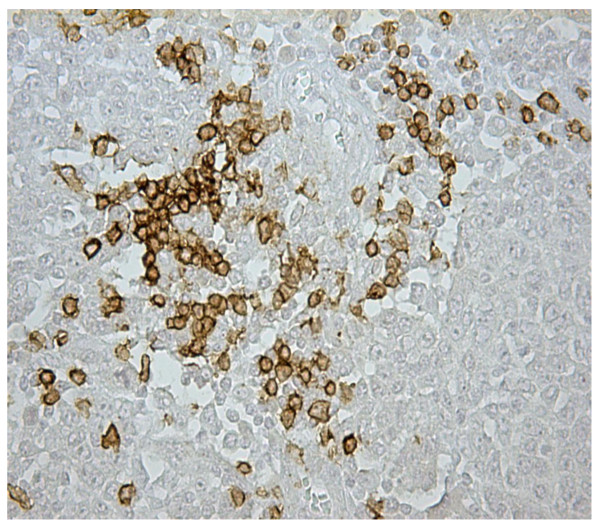
Immunostaining with keratin 20 (20×): the positivity is marked but not diffused.

## Discussion

Colorectal cancer is one of the most frequent oncological diseases. This clinical history shows a rare pattern of colon cancer metastasis. In patients with Dukes A, colon carcinoma relapse is rare [11]. Cutaneous or subcutaneous metastases are uncommon in colorectal cancer [1,2], as well as in carcinomas that have originated elsewhere [5,6,12,13].

The identification of this type of metastasis in many types of cancer is indicative of a poor prognosis with the presence of disseminated disease [5,6]. The study by Schoenlaub *et al*. reviewed 200 cases of cancers with cutaneous or subcutaneous metastasis and reported that among these, patients with colorectal cancer had a median survival of 4.4 months [5]. On the other hand, a retrospective study by Lookingbill *et al*. showed a median survival of 18 months in patients with the same characteristics [6].

A finding of isolated subcutaneous metastases of colorectal cancer without other metastatic sites is extremely rare and may delay the diagnosis by several months [14].

In our case, the rarity of this event caused many difficulties in the correct diagnosis and in the right therapeutic approach.

From a biological perspective, this case outlines the plural-phenotypic nature of cancer: the course of disease that we report here is clearly determined by a peculiar cancer cell population with specific biology and homing features.

## Conclusion

Despite the Dukes A staging, G2 grading and the absence of an increase in serum tumor markers, the characteristics of this cancer phenotype appear to be rather aggressive. Studies in molecular biology are strongly warranted to distinguish patients with a peculiar clinical course and prognosis. Intensive follow-up is also indicated in patients with good prognostic factors at diagnosis.

## Consent

Written informed consent was obtained from the patient for publication of this case report and any accompanying images. A copy of the written consent is available for review by the Editor-in-Chief of this journal.

## Competing interests

The authors declare that they have no competing interests.

## Authors’ contributions

GPS, FA, GLR, EM and ST designed the study, interpreted the study results and drafted the manuscript. FB contributed to the pathologic analysis. LC, VS, NP, SS and MS contributed to the work. All authors read and approved the final manuscript.
